# Changes in hemp secondary fiber production related to technical fiber variability revealed by light microscopy and attenuated total reflectance Fourier transform infrared spectroscopy

**DOI:** 10.1371/journal.pone.0179794

**Published:** 2017-06-22

**Authors:** Eva Fernandez-Tendero, Arnaud Day, Sandrine Legros, Anouck Habrant, Simon Hawkins, Brigitte Chabbert

**Affiliations:** 1 FARE Laboratory, INRA, Université de Reims Champagne-Ardenne, Reims, France; 2 Université de Lille, CNRS, UMR 8576 – UGSF - Unité de Glycobiologie Structurale et Fonctionnelle, Lille, France; 3 Fibres Recherche Développement, Troyes, France; 4 Terres Inovia, Troyes, France; Monash University, AUSTRALIA

## Abstract

Interest in hemp (*Cannabis sativa* L.) is increasing due to the development of a new range of industrial applications based on bast fibers. However the variability of bast fiber yield and quality represents an important barrier to further exploitation. Primary and secondary fiber content was examined in two commercial hemp varieties (Fedora 17, Santhica 27) grown under contrasted sowing density and irrigation conditions. Both growing conditions and hemp varieties impact stem tissue architecture with a large effect on the proportion of secondary fibers but not primary fibers. Attenuated total reflectance infrared spectroscopy allowed the discrimination of manually-isolated native primary fibers and secondary fibers but did not reveal any clustering according to growing conditions and variety. Infrared data were confirmed by wet chemistry analyses that revealed slight but significant differences between primary and secondary fiber cell wall composition. Infrared spectroscopy of technical fibers obtained after mechanical defibering revealed differences with native primary, but not secondary fibers and also discriminated samples obtained from plants grown under different conditions. Altogether the results suggested that the observed variability of hemp technical fibers could be partially explained by i) differences in secondary fiber production and ii) differential behavior during mechanical defibering resulting in unequal separation of primary and secondary fibers.

## Introduction

Hemp fibers have been traditionally used for centuries in the textile and paper industries [1]. Over the last few years, these natural fibers have also been increasingly used to replace synthetic fibers for the production of more environmentally friendly materials [1, 2]. In addition to their lower environmental impact, the use of natural fibers offers other advantages including lower density and good mechanical properties [3]. However, variability in the industrial quality represents an important barrier to the large-scale exploitation of these fibers. Previous studies have suggested that differences in the mechanical properties of fibers could be related to plant growth and the production of biological material [4, 5] as well as biological and/or physicochemical processing used for defibering and fiber purification [2, 6].

Hemp fibers are located in the outer stem tissues of the plant and are therefore referred to as bast fibers. Hemp bast fibers are in fact composed of two fiber types with different origins, primary bast fibers are sclerenchyma cells derived from the procambium whereas secondary bast fibers are produced by the vascular cambium [7]. These two fiber types show a number of differences in cell morphology and structure that could impact greatly on their suitability for different industrial uses. For example, the primary fibers with a greater cell length and higher crystalline cellulose content levels are preferred to secondary fibers for composite reinforcement [8, 9]. In practice, the separation of the two fiber types during the defibering process is difficult and secondary fibers remain attached to the primary fibers thereby affecting the overall quality of the harvested material [10–12]. The amount of secondary fibers varies according to the position in the plant stem (greater at the stem base) and increases with plant age [6, 9, 13]. The quantity of secondary fibers produced by hemp plants also depends upon the genotype (variety and sex) of the plant and is modified by contrasted growing conditions (environment) [10, 14–16]. Changes in growing conditions also affect the size and wall thickness of primary fibers [13, 15] that undergo significant chemical and structural changes from the vegetative to the seed maturity stage [6, 9, 17].

In addition to the fiber variability *in planta*, bast fiber quality and yield can be greatly modified by biological and/or physicochemical processing (retting, defibering, and all hydrothermal, chemical and enzymatic treatments that aim at minimizing damage during the mechanical separation of the fibers) [2, 6, 18–21]. In this context a reliable method for rapidly analyzing bast fibers would greatly facilitate optimization of downstream industrial processing and applications. Infrared spectroscopy has been extensively used for the characterization of plant cell walls because of its rapid sampling and high sensitivity [22, 23] and therefore represents a potentially interesting technique for characterizing hemp bast fibers. In hemp, Fourier transform infrared (FTIR) spectroscopy has been previously used to study the effect of enzymatic and chemical treatments, fiber dislocation and chemical composition [18, 24–26]. Another important parameter, cellulose crystallinity, can also be assessed by FTIR spectroscopy, although this parameter is usually better assessed by X-ray diffraction (XRD) approaches [24]. The development of the attenuated total reflectance technique (ATR) has also proved to be a useful approach by reducing sampling time and allowing non-destructive analysis of samples in their original environment [22].

The aim of this work was to evaluate the main sources of variability in fibers obtained from two hemp cultivars grown under different growing conditions using attenuated total reflectance—based infrared spectroscopy as a rapid and non-destructive tool.

## Material and methods

### Plant material

Hemp plants (*Cannabis sativa* L.) were grown at Marigny-le-Châtel, France (48°24’N, 3°44’E) by professional hemp producers. Field trials were conducted by the French Hemp Technical Institute ‘Terres Inovia’ (http://www.terresinovia.fr) that possesses all legal authorization and expertise necessary for hemp field studies. Two monoecïous fiber varieties (Fedora 17: F17 and Santhica 27: S27) were sown early April and cultivated under 3 different growing conditions on a chalky soil: Condition 1 (standard conditions): sowing density = 50 kg.ha^-1^, no irrigation; Condition 2 (irrigation): sowing density = 50 kg.ha^-1^ and irrigation corresponding to 50% additional water supply calculated on the base of average rainfall in this region (326 mm, MeteoFrance station Troyes–Barberey) and provided every 10 days during 3 months before flowering stage; Condition 3 (100 kg.ha^-1^): sowing density = 100 kg.ha^-1^, no irrigation. For all conditions, 120 U N.ha^-1^ ammonium nitrate was added under solid form at crop planting (one unit nitrogen correspond to 1 kg nitrogen provided by ammonium nitrate). Average rainfall was 319 mm for the cultivation year. Plants were harvested at the end of the flowering stage (17^th^ August and 22^nd^ August for F17 and S27, respectively).

### Microscopy

For microscopic observations of the stem basal regions (between 15 and 45 cm above ground level) were isolated from 4 individual plants for each growing condition and variety. Small discs (2.5 cm thick) were then removed and rapidly frozen in liquid nitrogen and stored at– 20°C. Frozen samples were gently thawed in deionized water at 4°C before hand-cutting thick transverse sections. The average thicknesses of bark (outer part of the stem) and wood (xylem, inner part of the stem) obtained were calculated from measurements made on thick transverse sections examined under a stereomicroscope (Stemi 2000-C, Zeiss, 30 measurements per section). The surface areas of primary and secondary fibers were determined using UV fluorescence microscopy (Axioskop, Zeiss equipped with filters at 340 nm excitation and 430 nm emission). Square images (715 X 715 μm) per section (4 samples per growing condition) were extracted and images analyzed with image-processing software (Axiovision 4, Zeiss and Image J).

### Fiber infrared analysis

Fibers were manually isolated from the basal stem region (between 15 and 45 cm above ground level) of individual plants. The bark of three plants per growing condition was separated from the inner core and fiber bundles were then peeled out and the epidermis discarded as previously described [9]. The secondary fibers were separated manually from primary ones under a stereomicrosope (Zeiss) without chemical treatment and native fiber bundles were then dried at room temperature. Three stem samples (1.5 kg) per variety/growing condition were also dried at 50°C in a forced-air oven and then mechanically defibered with a roller mill machine (Depoortere-Fibres Recherche Développement) to obtain technical fibers.

Manually-isolated and technical fibers were then characterized without grinding by infrared spectroscopy. Spectra were recorded by attenuated total reflectance (ATR) using a Nicolet 6700 spectrometer (ThermoScientific) equipped with a SmartPerformer ATR accessory. Measurements were made in triplicate on 3 samples per growing condition for each variety Spectra average were obtained from 64 scans recorded from 4,000 to 400 cm^-1^ at a resolution of 4 cm^-1^, and corrected for background absorbance by subtraction of the spectrum of the empty ATR crystal. FTIR spectra were baseline corrected and vector-normalized in the range of 1850–675 cm^-1^.

### Chemical analysis

Fiber bundles were ground with a ball crusher (Retsch, MM 2000) and extractives were removed using 80% ethanol at 40°C (20 ml/200mg, 4 times), then dried. Complete acid hydrolysis of cell wall polysaccharides into sugar monomers was performed using 1 M H_2_SO_4_ for 2 h at 100°C. The released monosaccharides were separated by high performance anion-exchange chromatography (PA1 column, Dionex) and analyses performed in triplicate as previously described [27]. Lignin content was estimated by a spectrophotometric procedure using acetyl bromide and 15 mg of cell wall residue (CWR) as previously described [28].

### Data analysis

Statistical analysis was performed with Statgraphics software. Equality of group variances was checked with a Barlett Test. The normality assumption was assessed by Kolmogrov Smirnov Test. Differences between treatments were evaluated using Analysis of Variance (ANOVA) and the HSD Tukey test used to separate averages at p < 0.005. When the normal curve of distribution was not verified or the number of samples was reduced, the non-parametric Kruskal Wallis test was applied. Simple regression analysis was used to show the correlation between certain variables. Multivariate statistical analysis of infrared spectra was performed by principal component analysis using the Unscrambler software (CAMO, Germany).

## Results and discussion

### Growing conditions affect bast fiber production

**Fig 1:**
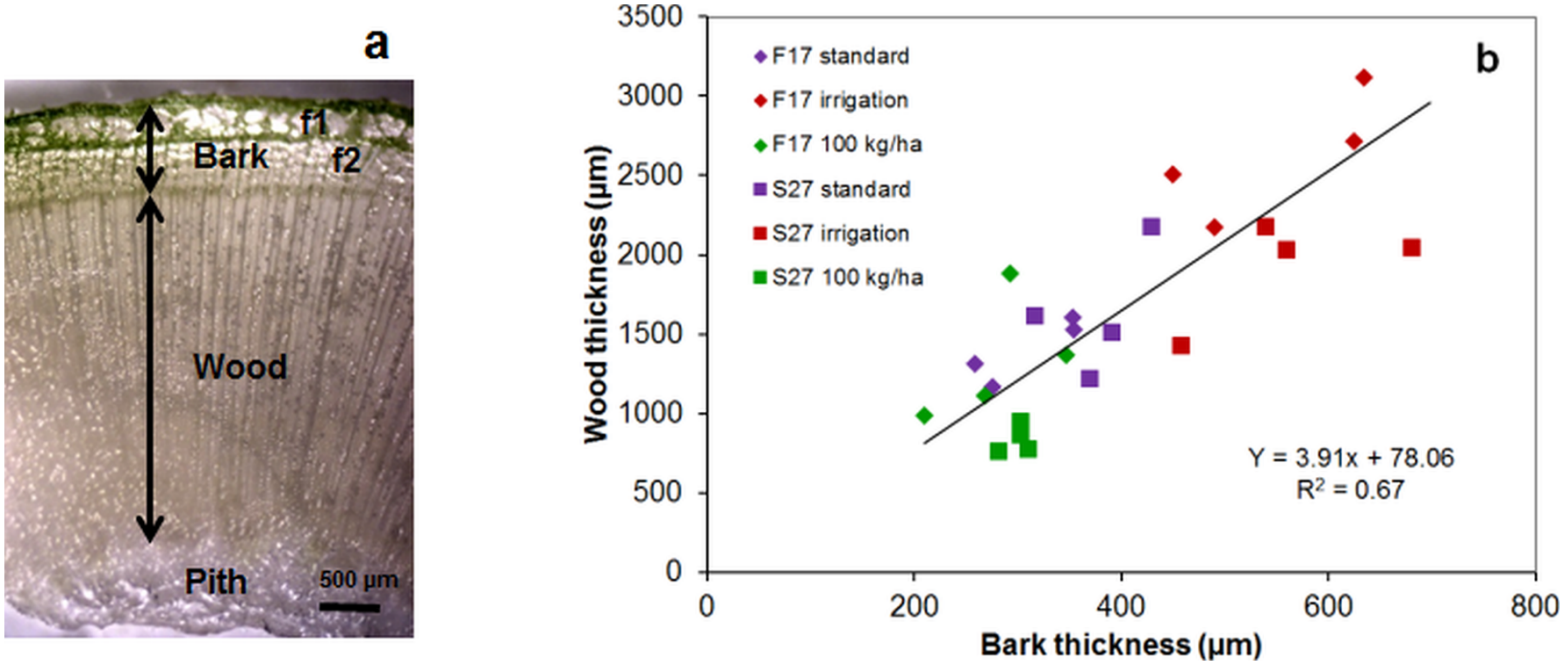
Stereomicroscopy imaging of transverse cross section of the hemp stem. (a) showing wood and bark including primary fibers (f1), secondary fibers (f2); arrows indicate the dimensions measured to determine wood-/bark-thickness (b) in the basal stem region of F17 and S27 grown under different conditions (standard, irrigation, high sowing density: 100 kg.ha^-1^).

Stem morphological features including bark and wood thickness and bast fiber distribution were determined by microscopy observations of hemp stem transverse sections. Examination of basal stem regions indicates significant variation in the thicknesses of stem outer tissues (designated as bark) and stem inner tissues (wood layer, but not pith) in transverse cross-sections (Fig 1a, S1 Table). For both varieties, the highest bark and wood thickness were obtained under irrigation conditions (p < 0.001) (Fig 1b). Overall the bark thickness shows a positive relation with the wood thickness and the different growing conditions did not significantly affect the overall bark/wood ratio for the two varieties (average ratio: F17 = 0.2, S27 = 0.3). Such an observation would suggest that changes in growing conditions equally affect bark and wood formation leading to significant changes in stem diameter, bark and wood thickness, but not the bark/wood ratio. One possible hypothesis to explain these results could be that the changes in growing conditions impact on the meristematic activity of the vascular cambium thereby modifying the number of derived cells differentiating into wood or secondary fibers in the bark. In agreement with this idea it is interesting to note that alterations in groundwater regime modify cambial activity and the number of cell derivatives in poplar, leading to changes in the rate of stem growth and circumference [29, 30]. Irrigation would favor individual plant growth giving rise to taller and thicker plants. In contrast, high seed sowing density has been reported to limit individual plant growth most likely as a result of inter-plant competition [16, 31]. Interestingly Schafer [15] observed that this reduction was more important in a dry year when compared to a wet year.

**Fig 2:**
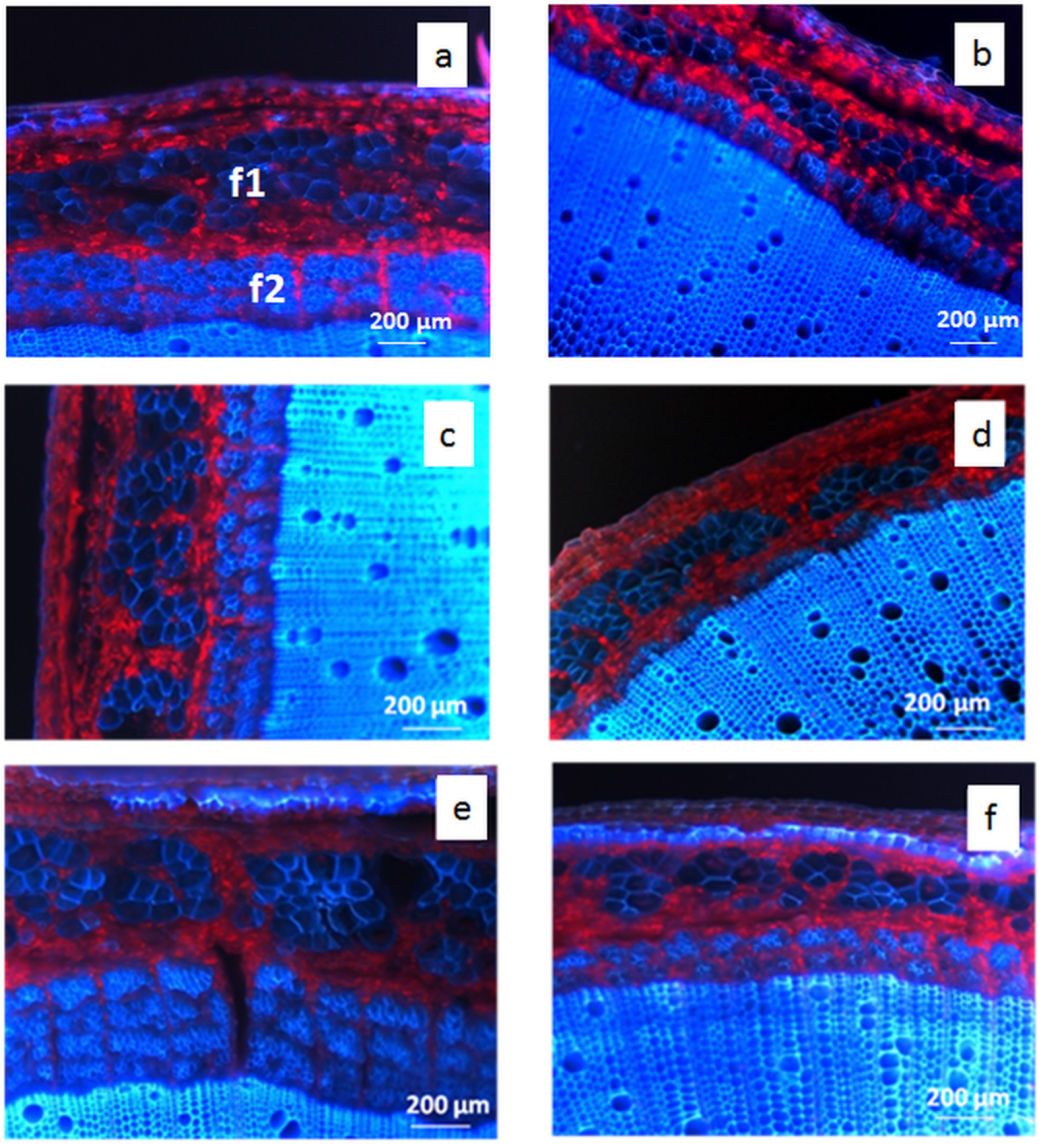
UV autofluorescence of cross-sections from the basal stem region of hemp. S27 (a,c,e) and F17 (b,d,f) grown under standard (a,b), high sowing density: 100 kg.ha^-1^ (c,d) irrigation (e,f). Primary fibers (f1), secondary fibers (f2).

Examination of the sections by UV fluorescence microscopy was then used to observe fiber distribution. UV autofluorescence which indicates the presence of lignin is essentially restricted to the middle lamella-primary cell wall region in both primary and secondary bast fibers, whereas autofluorescence is also detected in the secondary cell walls of xylem tissue [8, 9, 32] (Fig 2). Microscopic observations show that growing conditions impact mainly on the proportion of secondary bast fibers with high seed density (Fig 2c and 2d) and additional irrigation (Fig 2e and 2f) producing lesser and greater amounts of secondary fibers, respectively, when compared to standard conditions (Fig 2a and 2b) (S1 Table). In contrast to primary fibers, secondary fibers are present as several clearly defined bundle layers, the number of which increases with irrigation.

**Fig 3:**
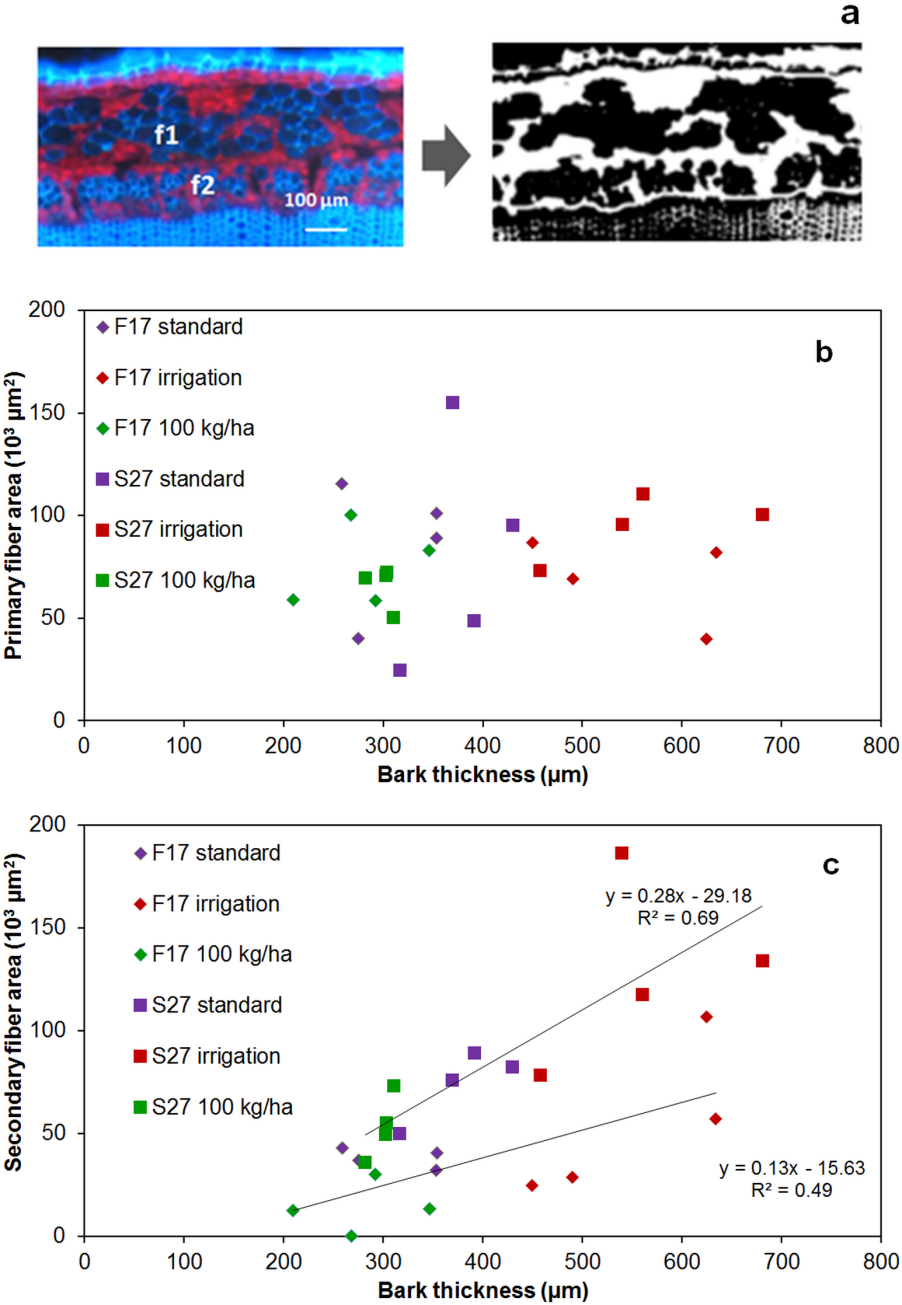
Image treatment of UV auto fluorescence of hemp stem cross-sections (a) used to determine areas of primary fibers (b) and secondary fibers (c) in F17 and S27 varieties grown under different conditions (standard, irrigation, high sowing density: 100 kg.ha^-1^) (expressed as μm^2^) with regards to bark thickness (expressed as μm).

Outer limits of fiber bundles were identified and used for image analysis to determine the areas corresponding to primary and secondary fibers thereby providing an overall indication of fiber quantity (Fig 3a). Results show that despite variations in bark thickness, growing conditions had no significant effect on the total area (cell wall plus lumen, expressed as μm^2^) of primary fibers (Fig 3b) and no significant differences were observed between the varieties. In contrast, the secondary fiber area significantly increased with additional irrigation (p<0.001) and was correlated with the bark thickness (Fig 3c) thereby supporting our hypothesis that cambial activity in hemp is dependent on growing conditions and variety. Such an observation is in agreement with previous studies suggesting that secondary fiber production decreases with increasing plant density [13, 15] and increases under rainy conditions [33]. The S27 variety contains more secondary fibers than F17 under all tested conditions (p < 0.001) indicating that plant genotype also affects secondary fiber development as previously reported [13, 34].

### ATR FTIR analysis of native fibers

**Fig 4:**
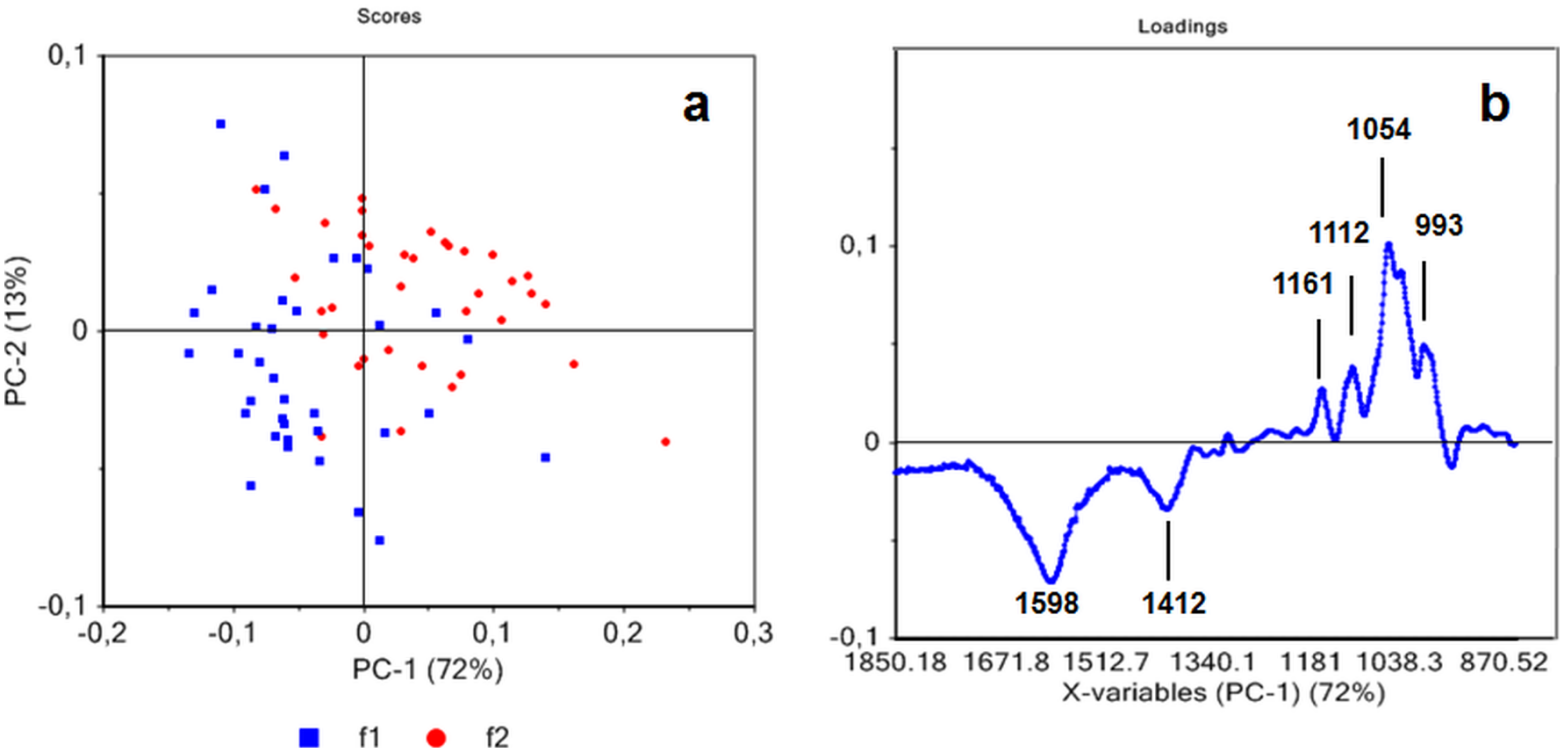
Principal component analysis of ATR spectra of hemp native primary fibers (f1) and secondary native fibers (f2) (a) obtained from F17 and S27 with all growing conditions. Score loading corresponding to PC1 (b).

Our results showed that the amount of secondary fibers produced depends upon both the genotype and the growing conditions thereby affecting the overall quality of the harvested material. To confirm that ATR-FTIR spectroscopy could be used as a potential tool for rapidly characterizing the proportions of primary and secondary fibers in hemp plants we firstly recorded spectra on manually-dissected primary and secondary fibers from both varieties grown under different conditions (S2 Table). Since the material used for ATR does not need any prior preparation the quantity required for analyses is greatly reduced. Principal component (PC) analysis indicates partial separation of the two fiber types (Fig 4a). The PC1 axis explains 72% of the statistically significant variation between samples showing that these results can be used to provide information about fiber type. Secondary fibers are positively correlated with PC1 positive loadings (Fig 4b) showing several peaks. The four peaks at 1161 (C-O-C asymmetric valence vibration), 1054 (C-O valence vibration), 993 (C-C, C-OH, C-H ring and side group vibrations) and 1112 cm^-1^ can be mainly attributed to cellulose and hemicellulose absorption [25, 35–37]. Negative loading from PC1 showed two main bands at 1598 cm^-1^ and 1412 cm^-1^ that can be assigned to the asymmetric and symmetric vibrations, respectively, of the COO (carboxylate) structure of esterified pectin [38]. Overall these results suggest that ATR FTIR is an effective tool for discriminating primary and secondary fibers based on the differential chemical composition of their cell walls.

**Fig 5:**
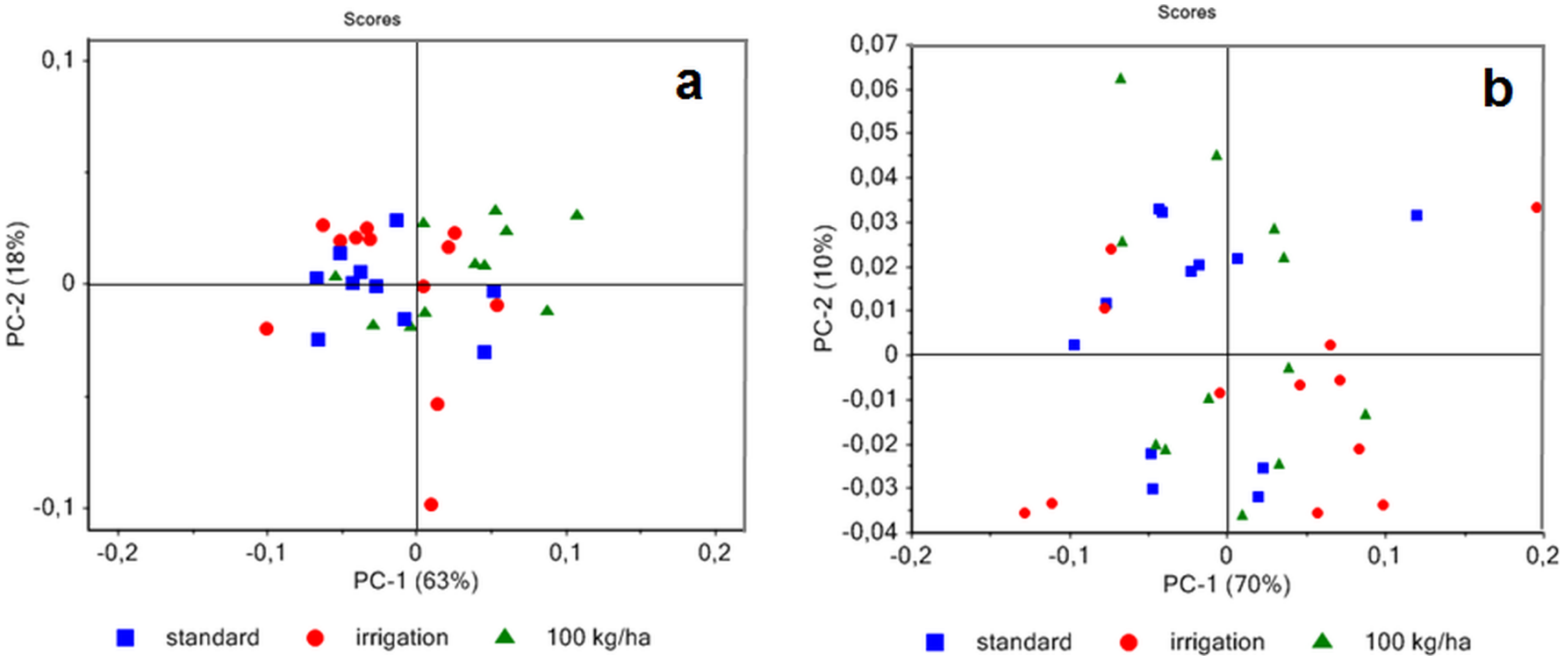
Principal component analysis of ATR spectra of native primary fibers (f1) (a) and of native secondary fibers (f2) (b) showing dispersion with regards to growing conditions.

When analyzed separately, no clear clustering of the spectra recorded on primary (Fig 5a) and secondary fibers (Fig 5b) could be observed according to the different growing conditions. At this stage of development (end of flowering stage) most of the structural components of the fiber cell wall have been deposited and our data therefore suggest that contrasted growing conditions do not strongly affect cell wall composition of both primary and secondary hemp fibers [6, 9].

### ATR FTIR analysis of technical fibers

**Fig 6:**
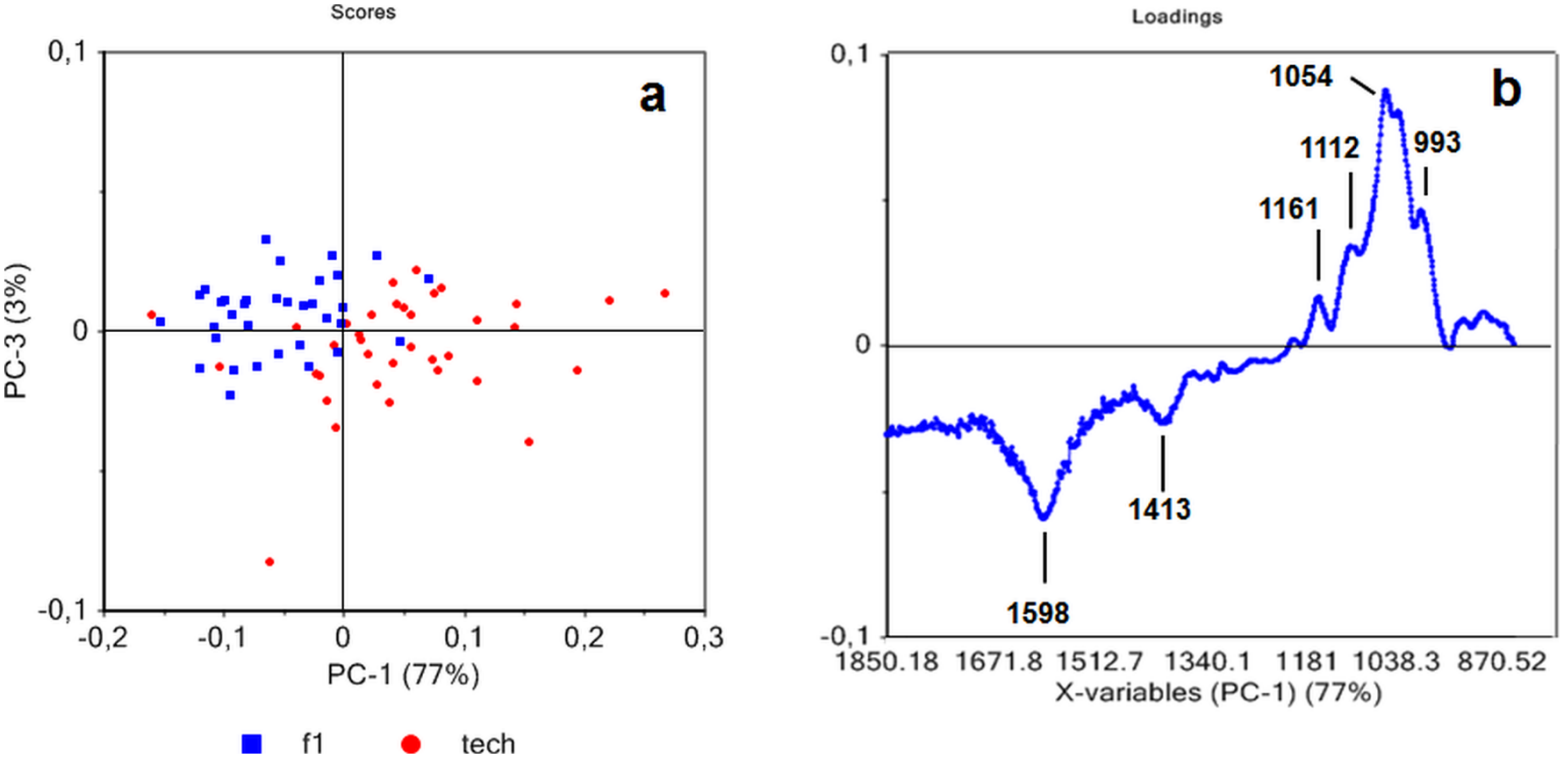
Principal component analysis of ATR spectra of hemp fibers showing dispersion with regards to technical fibers (tech) and native primary fibers (f1) (a). Score loading corresponding to PC1 (b).

**Fig 7:**
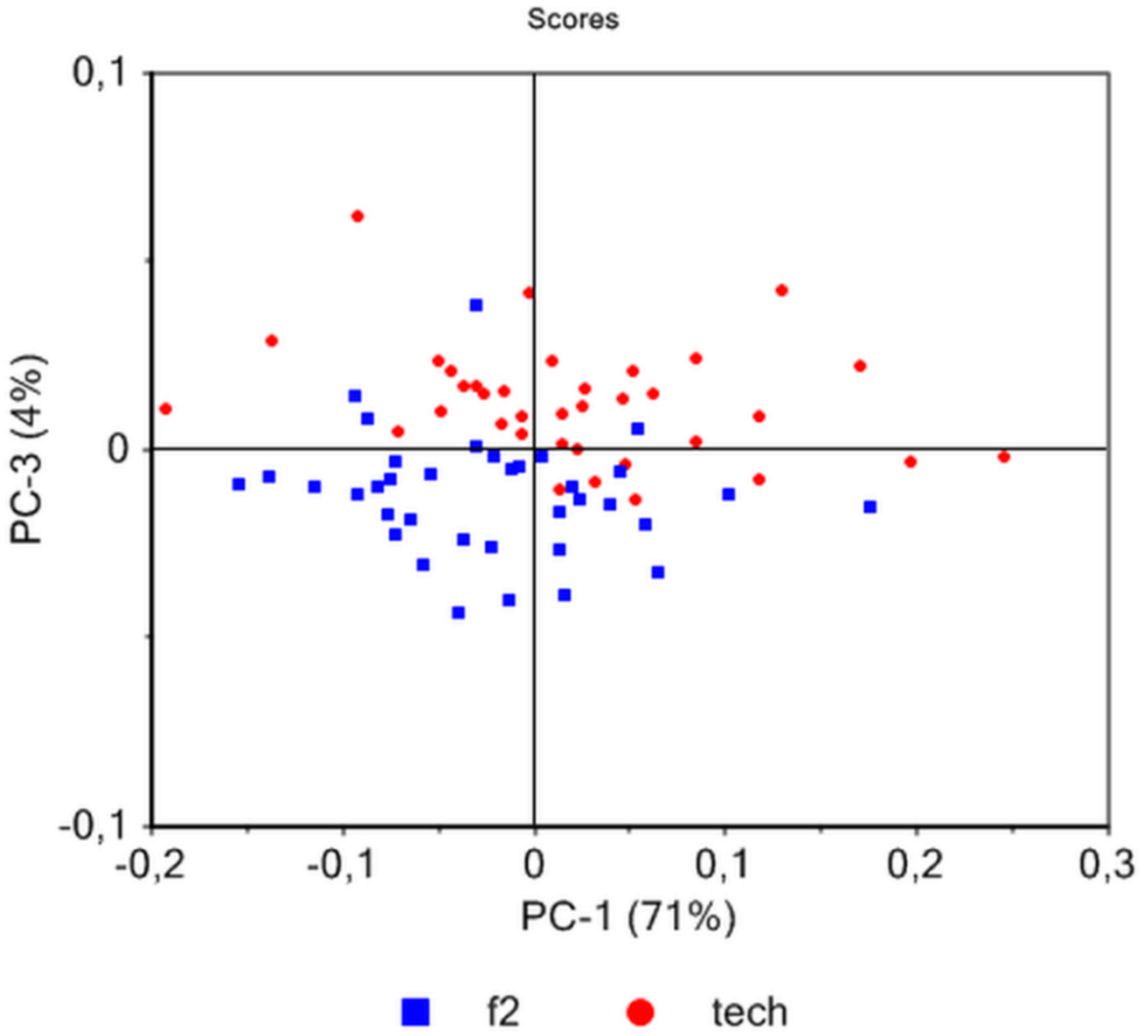
Principal component analysis of ATR spectra of native secondary and technical hemp fibers.

Since ATR FTIR successfully discriminated native primary and secondary fibers manually-isolated from the hemp stem we decided to see whether this technique could also be used to characterize technical fibers obtained by mechanical defibering of plants grown under the same conditions (S2 Table). We firstly compared ATR FTIR spectra of native primary and technical fiber samples obtained from plants grown under the same conditions. Principal component analysis indicates 77% of sample variation along the PC1 separating the majority of primary fibers and technical fibers (Fig 6a). The bands positively correlated with PC1 loadings are mainly related to technical fibers and include 4 peaks at 1161, 1112, 1054, 993 cm^-1^ (Fig 6b) that were related to secondary fibers in our previous analysis (Fig 4b). Two other bands at 1598 and 1413 cm^-1^ negatively correlated with the PC1 were also previously associated to primary fibers (Fig 4b). A similar comparison of spectra from native secondary and technical fiber samples (Fig 7) did not allow any significant separation of these two fiber types. Overall these results would suggest that technical fiber samples contain an important proportion of secondary fibers thereby suggesting that mechanical defibering can result in poor separation of these cell types.

**Fig 8:**
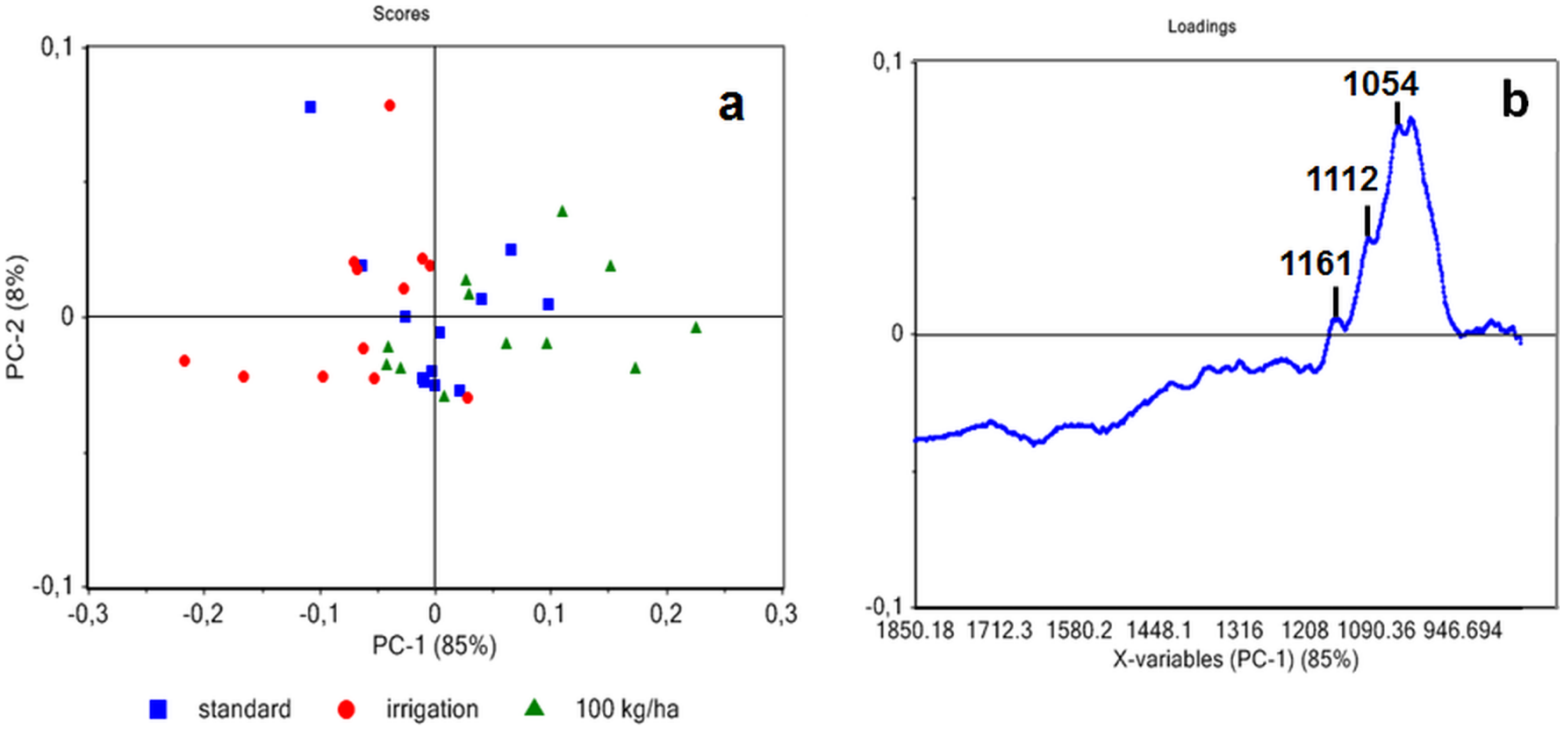
Principal component analysis of ATR spectra of technical fibers showing dispersion with regards to growing conditions (a). Score loading corresponding to PC1 (b).

Principal component analysis of just technical fibers (i.e. without native fibers) showed that spectra were separated along PC1 (Fig 8a). This component explained 85% variability and separates samples according to growing conditions, but not variety. No specific band was positively associated in the PC1 loading for samples obtained from plants grown under additional irrigation conditions whereas bands at 1161, 1112 and 1054 cm^-1^ were positively associated to technical fibers from high seed density samples (Fig 8b). These bands were also observed when native secondary fibers were compared to primary fibers (Fig 4b).

Altogether, these results would suggest that differences in secondary fiber content might partly explain the observed variability of the technical fibers obtained from hemp grown under different conditions. The absence of bands at 1161, 1112 and 1054 cm^-1^ in the PC loading of ATR FTIR spectra of technical fibers obtained from plants grown under additional irrigation conditions would suggest that few/no secondary fibers are present. These results are in clear contradiction with our microscopic observations showing that greater amounts of secondary fibers are present in plants grown under additional irrigation conditions as compared to plants grown under high density conditions (Fig 3b). One possible explanation for this unexpected result is that growing conditions may affect mechanical behavior during defibering. Secondary fibers might remain attached to primary fibers in technical fiber samples obtained from plants grown under high seed density conditions. In contrast, they become separated from primary fibers in samples obtained from plants grown with additional irrigation. This hypothesis is supported by measurements (Table 1) showing that the proportion of technical fibers as a percentage of straw yields is slightly lowest for plants grown under additional irrigation (both varieties). In this scenario, although additional irrigation results in the production of higher amounts of secondary fibers, these fibers are more easily separated from primary fibers leading to i) reduced total fiber yield and ii) absence of secondary fiber ATR FTIR bands in technical fiber samples produced by mechanical defibering. Further analyses of technical fibers should allow clarification of this point and, if confirmed, would suggest that the presence/absence of secondary fibers represent an important source of the observed variability between technical fibers samples. Cell-cell cohesion in plants depends upon the structure of the middle lamella and it would obviously be interesting to see whether the observed differences in primary-secondary fiber separation are related to differences in outer stem morphology (e.g. quantity of parenchyma cells between the two fiber types), or to modifications in the middle lamella structure.

**Table 1:** Fiber yields of F17 and S27 hemp varieties harvested at the end of flowering stage (as percent of straw dry matter).

Variety	Standard	Irrigation	100 kg.ha^-1^
F17	27.7 ± 0.6	26.5 ± 0.8	28.1 ± 0.2
S27	32.4 ± 0.7	31.3 ± 0.1	32.9 ± 0.1

Overall our results indicate that the main difference in technical fiber quality appears to be based upon the relative proportions of primary and secondary fibers. In order to complete this survey of hemp bast fiber content we also determined the structure of manually dissected primary and secondary fibers by wet chemistry. For this we used the S27 variety that contained significantly higher (and sufficient for analysis) amounts of secondary fibers (Fig 3b). The results showed that lignin content in secondary fibers was significantly higher (5.5% on average) than in primary fibers (3.6% on average), (p < 0.001) (Table 2). Sulfuric acid hydrolysis also revealed that secondary fibers polysaccharides contained significantly more xylose (p < 0.001). Albeit weak these variations in chemical composition between primary and secondary fibers suggest that cell wall architecture differs slightly between the two types of fibers. Except for the proportion of mannose in secondary fibers (significant decrease under high seed density conditions), wet chemistry did not reveal any large variations in cell wall composition between plants grown under different growth conditions in agreement with infrared analysis.

**Table 2:** Chemical composition of primary and secondary fibers isolated from hemp harvested at the end of flowering stage (variety S27). Lignin and total sugar contents as percent of cell wall residue; monosaccharides as percent of total sugars. Means not sharing a common letter are significantly different (p<0.05).

Fibers	Growing conditions	Lignin	Total Sugars	Glc	Gal	Xyl	Man	GalU
Primary	Standard	3.0 ^a^	79.9 ^a^	90.1 ^a^	2.5 ^a^	0.2 ^a^	5.5 ^a^	4.1 ^a^
	Irrigation	3.0 ^a^	74.7 ^a^	90.0 ^a^	2.7 ^a^	0.3 ^a^	5.4 ^a^	3.9 ^a^
	100 kg.ha^-1^	3.4 ^a^	75.0 ^a^	91.1 ^a^	2.2 ^a^	0.3 ^a^	4.7 ^a^	3.5 ^a^
Secondary	Standard	5.5 ^a^	72.3 ^a^	87.1^a^	2.9 ^a^	1.8 ^b^	6.2 ^a^	4.4 ^a^
	Irrigation	5.5 ^a^	70.1 ^a^	86.0 ^a^	3.1 ^a^	2.8 ^b^	5.7 ^a^	4.4 ^a^
	100 kg.ha^-1^	5.6 ^a^	67.7 ^a^	90.4 ^a^	2.6 ^a^	1.9 ^b^	2.9 ^b^	4.8 ^a^

## Conclusion

Variability in technical fiber quality is generally considered as one of the major disadvantages of natural (plant) fibers and represents an important obstacle to their widespread use in composite materials. A rapid and reliable method of evaluating technical fiber quality would therefore represent a valuable help to industry. In this paper we have shown that ATR FTIR spectroscopy represents an efficient and powerful technique for evaluating variability in technical fibers obtained from different sources. Both growing conditions and genetic differences (variety effect) impact on hemp stem tissue architecture with a major effect on i) secondary (but not primary) fiber production and ii) primary and secondary fiber separation during mechanical defibering. Our results also indicate that a major source of variability in technical fibers is related to the presence of secondary fibers. This information represents an important contribution to our understanding of the biological reasons underlining the variability of natural fibers and should lead to the improvement of production and defibering protocols necessary for a better control of fiber quality. The use of ATR infrared analysis also provides chemical information about the surfaces of technical fibers. Such information is of great importance for a better understanding of bast fiber cohesion in agrocomposites and is complementary to information about the mechanical properties of technical fibers.

## Supporting information

S1 Table Thickness of stem tissues and surfaces of fibers. (XLSX)

S2 Table Infrared analysis of hemp fibers. (XLSX)

## References

[1] Ranalli P. Current status and future scenarios of hemp breeding. Euphytica. 2004;140(1–2):121–31.

[2] Faruk O, Bledzki AK, Fink H-P, Sain M. Biocomposites reinforced with natural fibers: 2000–2010. Progress in Polymer Science. 2012;37(11): 1552–96.

[3] Joshi CP, Bhandari S, Ranjan P, Kalluri UC, Liang X, Fujino T, et al Genomics of cellulose biosynthesis in poplars. New Phytologist. 2004;164(1):53–61. 10.1111/j.1469-8137.2004.01155.x 33873484

[4] Thygesen LG. The effects of growth conditions and of processing into yarn on dislocations in hemp fibres. Journal of Materials Science. 2011;46(7):2135–9.

[5] Abot A, Bonnafous C, Touchard F, Thibault F, Chocinski-Arnault L, Lemoine R, et al Effects of cultural conditions on the hemp (*Cannabis sativa*) phloem fibres: Biological development and mechanical properties. Journal of Composite Materials. 2013;47(8):1067–77.

[6] Liu M, Fernando D, Daniel G, Madsen B, Meyer AS, Ale MT, et al Effect of harvest time and field retting duration on the chemical composition, morphology and mechanical properties of hemp fibers. Industrial Crops and Products. 2015;69(0):29–39.

[7] Gorshkova T, Brutch N, Chabbert B, Deyholos M, Hayashi T, Lev-Yadun S, et al Plant fiber formation: state of the art, recent and expected progress, and open questions. Critical Reviews in Plant Sciences. 2012;31(3):201–28.

[8] Bonatti PM, Ferrari C, Focher B, Grippo C, Torri G, Cosentino C. Histochemical and supramolecular studies in determining quality of hemp fibres for textile applications. Euphytica. 2004;140(1–2):55–64.

[9] Crônier D, Monties B, Chabbert B. Structure and chemical composition of bast fibers isolated from developing hemp stem. Journal of Agricultural and Food Chemistry. 2005;53(21):8279–89. doi: 10.1021/jf051253k 16218676 10.1021/jf051253k

[10] Mediavilla V, Leupin M, Keller A. Influence of the growth stage of industrial hemp on the yield formation in relation to certain fibre quality traits. Industrial Crops and Products. 2001;13(1):49–56.

[11] Pickering KL, Li Y, Farrell RL, Lay M. Interfacial modification of hemp fiber reinforced composites using fungal and alkali treatment. Journal of Biobased Materials and Bioenergy. 2007;1(1):109–17.

[12] Placet V, Meteau J, Froehly L, Salut R, Boubakar ML. Investigation of the internal structure of hemp fibres using optical coherence tomography and Focused Ion Beam transverse cutting. Journal of Materials Science. 2014;49(24):8317–27.

[13] Amaducci S, Zatta A, Pelatti F, Venturi G. Influence of agronomic factors on yield and quality of hemp (*Cannabis sativa* L.) fibre and implication for an innovative production system. Field Crops Research. 2008;107(2):161–9.

[14] Sankari HS. Comparison of bast fibre yield and mechanical fibre properties of hemp (*Cannabis sativa* L.) cultivars. Industrial Crops and Products. 2000;11:73–84.

[15] Schäfer T, Honermeier B. Effect of sowing date and plant density on the cell morphology of hemp (*Cannabis sativa* L.). Industrial Crops and Products. 2006;23(1):88–98.

[16] Westerhuis W, Amaducci S, Struik PC, Zatta A, Van Dam JEG, Stomph TJ. Sowing density and harvest time affect fibre content in hemp (*Cannabis sativa*) through their effects on stem weight. Annals of Applied Biology. 2009;155(2):225–44.

[17] Blake AW, Marcus SE, Copeland JE, Blackburn RS, Knox JP. In situ analysis of cell wall polymers associated with phloem fibre cells in stems of hemp, *Cannabis sativa* L. Planta. 2008;228(1):1–13. doi: 10.1007/s00425-008-0713-5 18299887 10.1007/s00425-008-0713-5

[18] Ouajai S, Shanks RA. Morphology and structure of hemp fibre after bioscouring. Macromolecular Bioscience. 2005;5(2):124–34. doi: 10.1002/mabi.200400151 15719429 10.1002/mabi.200400151

[19] Islam MS, Pickering KL, Foreman NJ. Influence of hygrothermal ageing on the physico-mechanical properties of alkali treated industrial hemp fibre reinforced polylactic acid composites. Journal of Polymers and the Environment. 2010;18(4):696–704.

[20] Kostic MM, Pejic BM, Asanovic KA, Aleksic VM, Skundric PD. Effect of hemicelluloses and lignin on the sorption and electric properties of hemp fibers. Industrial Crops and Products. 2010;32(2):169–74.

[21] Hänninen T, Thygesen A, Mehmood S, Madsen B, Hughes M. Mechanical processing of bast fibres: The occurrence of damage and its effect on fibre structure. Industrial Crops and Products. 2012;39(0):7–11.

[22] Abidi N, Cabrales L, Haigler CH. Changes in the cell wall and cellulose content of developing cotton fibers investigated by FTIR spectroscopy. Carbohydrate Polymers. 2014;100:9–16. doi: 10.1016/j.carbpol.2013.01.074 24188832 10.1016/j.carbpol.2013.01.074

[23] Lupoi JS, Singh S, Simmons BA, Henry RJ. Assessment of lignocellulosic biomass using analytical spectroscopy: an evolution to high-throughput techniques. Bioenergy Research. 2014;7(1):1–23.

[24] Le Troedec M, Sedan D, Peyratout C, Bonnet JP, Smith A, Guinebretiere R, et al Influence of various chemical treatments on the composition and structure of hemp fibres. Composites Part A-Applied Science and Manufacturing. 2008;39(3):514–22.

[25] Dai D, Fan M. Investigation of the dislocation of natural fibres by Fourier-transform infrared spectroscopy. Vibrational Spectroscopy. 2011;55(2):300–6.

[26] Kabir MM, Wang H, Lau KT, Cardona F, Aravinthan T. Mechanical properties of chemically-treated hemp fibre reinforced sandwich composites. Composites Part B-Engineering. 2012;43(2):159–69.

[27] Beaugrand J, Chambat G, Whong VWK, Goubet F, Rémond C, Paës G, et al Impact and efficiencey of GH10 and GH11 thermostable endoxylanases on wheat bran and alkali-extractable arabinoxylans. Carbohydrate Research. 2004;339:2529–40. doi: 10.1016/j.carres.2004.08.012 15476714 10.1016/j.carres.2004.08.012

[28] Iiyama K, Wallis AFA. Determination of lignin in herbaceous plants by an improved acetyl bromide procedure. Journal of the Science and Food Agriculture. 1990;51:145–61.

[29] Arend M, Fromm J. Seasonal change in the drought response of wood cell development in poplar. Tree Physiology. 2007;27(7):985–92. 17403651 10.1093/treephys/27.7.985

[30] Berta M, Giovannelli A, Potenza E, Traversi ML, Racchi ML. Type 3 metallothioneins respond to water deficit in leaf and in the cambial zone of white poplar (*Populus alba*). Journal of Plant Physiology. 2009;166(5):521–30. doi: 10.1016/j.jplph.2008.08.005 18845361 10.1016/j.jplph.2008.08.005

[31] Amaducci S, Amaducci MT, Benati R, Venturi G. Crop yield and quality parameters of four annual fibre crops (hemp, kenaf, maize and sorghum) in the North of Italy. Industrial Crops and Products. 2000;11(2–3):179–86.

[32] Liu M, Fernando D, Meyer AS, Madsen B, Daniel G, Thygesen A. Characterization and biological depectinization of hemp fibers originating from different stem sections. Industrial Crops and Products. 2015;76:880–91.

[33] Hoppner F, Mange-Hartmann U. Cultivation experiments with two fibre hemp varieties. Journal of the International Hemp Association. 1995;2(1):18–22.

[34] Mediavilla V, Bassetti P, Leupin M, Mosimann E. Agronomic characteristics of hemp varieties. Agrarfoschung. 1999;6(10):393–6.

[35] Faix O. Classification of lignins from different botanical origins by FT-IR spectroscopy. Holzforschung. 1991;45:21–7.

[36] Schwanninger M, Rodrigues J, Pereira H, Hinterstoisser B. Effects of short-time vibratory ball milling on the shape of FT-IR spectra of wood and cellulose. Vibrational Spectroscopy. 2004;36(1):23–40.

[37] Akerholm M, Salmen L. Interactions between wood polymers studied by dynamic FT-IR spectroscopy. Polymer. 2001;42:963–9.

[38] Santos JDG, Espeleta AF, Branco A, de Assis SA. Aqueous extraction of pectin from sisal waste. Carbohydrate Polymers. 2013;92(2):1997–2001. doi: 10.1016/j.carbpol.2012.11.089 23399250 10.1016/j.carbpol.2012.11.089

